# Pain trajectories and possible predictors of a favourable course of low back pain in patients consulting musculoskeletal physicians in The Netherlands

**DOI:** 10.1186/s12998-021-00392-3

**Published:** 2021-09-22

**Authors:** Wouter Schuller, Raymond W. Ostelo, Daphne C. Rohrich, Martijn W. Heymans, Henrica C. W. de Vet

**Affiliations:** 1 Amsterdam UMC, Location VUmc, Department of Data Science and Biostatistics, Amsterdam Public Health Research Institute, de Boelelaan 1117, Amsterdam, The Netherlands; 2 Spine Clinic, Provincialeweg 152, 1506 ME, Zaandam, The Netherlands; 3 Department of Health Science, Faculty of Science, VU University, and Amsterdam Movement Sciences, de Boelelaan 1105, 1081 HV Amsterdam, The Netherlands

**Keywords:** Low back pain, Spinal manipulative treatment, Musculoskeletal medicine, Pain trajectories, Prediction model

## Abstract

**Background:**

In The Netherlands, low back pain patients can consult physicians specialized in musculoskeletal (MSK) medicine. Previous studies have reported on the characteristics of patients consulting MSK physicians, and the treatment options used. There are no studies yet reporting on the course of Low Back Pain (LBP) after treatment by musculoskeletal (MSK) physicians in The Netherlands.

**Methods:**

In an observational cohort study MSK physicians recorded data about all low back pain patients presenting for a first consultation. At baseline they recorded age, gender, type and duration of the main complaint, and concomitant complaints. At the end of treatment they recorded the type of treatment and the number of treatment sessions. Patients were recruited to answer questionnaires at baseline, and at 6-weekly intervals during a follow-up period of six months. Patient questionnaires included information about previous medical consumption, together with PROMs measuring the level of pain and functional status. Latent Class Growth Analysis (LCGA) was used to classify patients into different groups according to their pain trajectories. Baseline variables were evaluated as predictors of a favourable trajectory using logistic regression analyses, and treatment variables were evaluated as possible confounders.

**Results:**

A total of 1377 patients were recruited, of whom 1117 patients (81%) answered at least one follow-up measurement. LCGA identified three groups of patients with distinct pain trajectories. A first group (N = 226) with high pain levels showed no improvement, a second group (N = 578) with high pain levels showed strong improvement, and a third group (N = 313) with mild pain levels showed moderate improvement. The two groups of patients presenting with high baseline pain scores were compared, and a multivariable model was constructed with possible predictors of a favourable course. Male gender, previous specialist visit, previous pain clinic visit, having work, a shorter duration of the current episode, and a longer time since the complaints first started were predictors of a favourable course. The multivariable model showed a moderate area under the curve (0.68) and a low explained variance (0.09).

**Conclusions:**

In low back pain patients treated by musculoskeletal physicians in The Netherlands three different pain trajectories were identified. Baseline variables were of limited value in predicting a favourable course.

**Supplementary Information:**

The online version contains supplementary material available at 10.1186/s12998-021-00392-3.

## Background

Low back pain (LBP) is a major health problem, with a point prevalence in Western countries of 12–30% [1]. In 2010, in The Netherlands, total costs were estimated to be €3.5 billion, including both direct costs, related to the consumption of medical care, and indirect costs, related to loss of productivity and disability pensions [1]. Because in most cases the mechanism of LBP is not known, there is no intervention that can be directed at the cause of the pain, and while many interventions are available, none has shown to be superior [2]. Although the course of low back pain has long been considered favourable, recurrences are common [3, 4], and many patients (65%) still reported pain 1 year after onset [5]. Considering the recurrent character of LBP, recent research has increasingly focused on identifying LBP trajectories [6, 7]. Distinct clusters of pain trajectories were identified [8], and over the course of their LBP, patients showed consistent cluster membership [6]. Rather than studying prognostic factors based upon outcome measurements at one follow-up moment, it may therefore be more informative to follow patients for longer periods of time, and to identify prognostic factors that predict the trajectory of LBP. This knowledge can potentially be used in outcome research, studying whether interventions can influence patients pain trajectories, rather than offering momentary improvement [9]. Measuring pain trajectories has become easier with the development of automated systems distributing patient reported questionnaires over the internet, or by using text messages. A recent study by Ailliet et al., for example, used text messages to study the pain trajectories of patients with low back and neck pain in patients consulting chiropractors in The Netherlands and Belgium [10]. A review of LBP trajectory studies by Kongsted et al. stated that it would be highly relevant to investigate whether LBP trajectories identify phenotypes of LBP that benefit from different care pathways [11].

In The Netherlands, among the various diagnostic and treatment possibilities, patients can consult physicians specialized in Musculoskeletal Medicine (MSK). MSK physicians are generally consulted by patients with musculoskeletal pain, and about half of the patients consult MSK physicians because of LBP [12]. MSK physicians use an array of diagnostic and treatment options, almost invariably including a form of Spinal Manipulative Therapy (SMT). The aim of our study was to assess whether different pain trajectories could be identified in LBP patients after consulting MSK physicians, and to identify possible predictors of a favourable course.

## Methods

### Study design and participants

We conducted a prospective cohort study with a follow-up period of six months.

All MSK physicians registered with the Dutch Association for Musculoskeletal Medicine were invited to participate in our study. Participating physicians were instructed to register all patients who presented for the first time in an MSK practice through a web-based registry. Patients were invited to take part in the study when making a first appointment. Inclusion criteria were LBP, age ≥ 18, and sufficient mastery of the Dutch language to answer questionnaires in Dutch. If patients gave informed consent, the treating physician entered email addresses of the recruited patients in the web-based registry. Thereafter, a specially designed computer program (Readmail) was used to automatically distribute invitations to patients by email to fill in web-based questionnaires.

### Study procedure

Both the treating physicians and the individual patients provided data via web-based registries. The treating physicians recorded data at baseline and at the end of the treatment. Study procedures were explained to participating physicians during specially organized information sessions. In addition, a research assistant visited all participating practices to explain the procedures. Practices that agreed to participate at a later stage were informed by telephone. Instructions were to ask all consecutive patients presented for a first consultation to participate in the study. Recruited patients received invitations to fill in web-based questionnaires within three weeks before the first consultation, and at six weekly intervals during the ensuing six months. When patients did not respond, a maximum of three reminders were sent within a period of two weeks. Both the invitation email and the web-based questionnaires contained links to a leaflet with information about the study.

### Measurement

At baseline physicians registered data about age, gender, type and duration of the main complaint, and the existence of concomitant complaints. Complaints were registered according to the International Classification of Primary Care (ICPC) [13]. At the end of treatment, data were registered about the number of treatment sessions, the type of treatment used, and further referrals.

At baseline, patients were asked to indicate whether their main complaint was low back pain, neck pain or any other complaint. This question was supported by text and manikins, explaining which area was considered to cover neck pain or low back pain. For other complaints, patients could explain these in text. Patients were asked to indicate whether their pain radiated to the legs or arms, and whether they had numbness or pins and needles in their legs or arms. Patients were also asked about the duration of the current episode, the time since the first episode, educational level, work status, previous specialist consultations, and previous treatments. The effect of previous treatments was measured on an ordinal scale, with four possible answer categories; 1.strong improvement, 2.little improvement, 3.unchanged, 4.deteriorated. Furthermore, all patients were asked to answer a set of Patient Reported Outcome Measures (PROMs), including a Numerical Rating Scale (NRS) for pain severity, the SF6D [14], and the Fear Avoidance Beliefs Questionnaire (FABQ) [15, 16]. Patients who indicated LBP as their main complaint were asked to answer the Oswestry Disability Index (ODI) [17, 18]. The SF6D is a short version of the SF36, measuring health related quality of life. Scores range from 0–1, with higher scores indicating lower quality of life. The FABQ consists of 16 items, and measures pain related fear in LBP patients. Higher scores indicate more pain related fear. The ODI consists of 10 items with scores ranging from 0–50, with higher scores indicating more disability because of LBP. At all follow-up points patients were asked to answer the same PROMs, except for the FABQ. A question about the Global Perceived Effect (GPE) of treatment was added.

### Statistical analyses

#### Identification of pain trajectories

Our study population consisted of all LBP patients who completed the baseline questionnaire. For the analyses of pain trajectories, patients were selected who completed the baseline questionnaire and at least one of the follow-up questionnaires. Latent Class Growth Analyses (LCGA) were used to explore whether subgroups of patients following distinct pain trajectories could be identified, using the NRS for pain scores [19]. Several LCGA models were evaluated with different numbers of trajectories, allowing linear or quadratic pain trajectories, and allowing more or less heterogeneity in pain trajectories within subgroups. For competing models the posterior probabilities were compared (average, range and standard deviation of the latent class probability per class). A final model was selected based upon model fit and considerations of interpretability and clinical practicality, proportion of patients in each class, and the final outcome after a follow-up of six months. Model fit was evaluated with the Vuong-Lo-Mendell-Rubin likelihood ratio test (LMR-LRT) and the Bayesian Information Criterion (BIC) [20]. The BIC considers both the likelihood of the model as well as the number of parameters in the model, with lower values showing better model fit. The LMR-LRT provides a p-value. A significant p-value indicates that a model with k classes fits better than a model with k-1 classes. LCGA was carried out using Mplus (Version 7) [20, 21].

#### Identifying possible predictors

Descriptive analyses of baseline variables were carried out for the complete population included in the analyses, and for each group of patients with a distinct pain trajectory separately. For the patients that presented with high baseline pain scores two distinct pain trajectories were identified (see results). One trajectory identified a group of patients who did not improve (class 1), and one trajectory identified a group of patients who improved (class 2). Logistic regression analyses were conducted to study the univariate relationship between baseline variables and the dependent variable (i.e. high baseline pain and not improved (class 1) vs high baseline pain and improved (class 2)).

A backward selection procedure was carried out on cases with complete data on all variables to construct a multivariable model, based upon a p-value of 0.157 (Akaike criterion). Treatment variables were considered as possible confounders instead of predictors. Although not known at baseline, they could possibly influence the outcome. To evaluate the influence of treatment variables the backwards selection was therefore carried out twice, with and without including treatment variables (type of treatment, number of treatment sessions, and adjuvant McKenzie treatment). The relationship between continuous predictors and the outcome was tested for linearity, and non-linear variables were entered as splines. The fit of the final model was evaluated with the loglikelihood and the Hosmer Lemeshow test [22]. Discriminative properties of the model were evaluated by calculating the Area Under the Curve (AUC), and Nagelkerke R^2^ [23] was used as an overall measure to quantify the amount of information in the model. Bootstrapping was used for internal validation [24]. Descriptive analyses and univariate analyses were carried out using SPSS 22, except for the univariate analyses of non-linear variables. Linearity, univariate analyses of non-linear variables and internal bootstrap validation was carried out with the R-package rms (version 5.1–2). In the multivariable analyses, backwards selection was carried out with the R-package pfsmi [25].

### Missing data and evaluation of loss to follow-up

The relationship between complete predictors and predictors with missing values (> 20%) was studied with univariate logistic regression analyses. In this analysis, significant relationships between predictors and the variable being either missing or not missing support the assumption that missing values are probably missing at random (MAR). When including all potential predictors in the model, the percentage of missing cases was around 40%, which required 40 multiple imputed datasets. Multivariable analyses were conducted in each dataset, and results were pooled according to Rubin`s rules [26]. To evaluate the influence of loss to follow-up, the group of patients who only answered the baseline questionnaires was compared with the group of patients answering at least one follow-up measurement. Differences in baseline characteristics between these two groups were studied with logistic regression. Multiple imputation and evaluation of missing data were carried out using SPSS 22.

## Results

### Study population

Data was collected from February 2014 until February 2016. A discrepancy was found between the number of patients classified with low back pain by the physician, and the self-classification by the patient through the web-based questionnaire. Frequently, patients classified themselves as other, but indicated complaints in text that would classify as low back pain. It was therefore decided to use the classification of the physician to select LBP patients. In the web-based registry MSK physicians recorded 2026 patients with LBP. Of these patients 1664 were recruited for our study. Our study population consisted of 1377 patients who answered the baseline questionnaire. A total of 1117 patients (81%) answered at least one of the follow up measures next to the baseline questionnaire and were included in the LCGA and prediction analyses. Of these 1117 patients, 93% answered the first follow-up questionnaire, 74% the second, 58% the third, and 43% the fourth. Although percentages missing increased over time, patients frequently missed intermediate questionnaires. Baseline characteristics of the whole sample, the patients who answered at least one follow-up questionnaire and were included in the analyses, and the patients who were lost to follow-up are presented in Table1. Although 19 practices participated in the study, the LBP patients were recruited by 16 practices, and the number of LBP patients recruited by the various practices varied from 1–285.

**Table 1:** Sample characteristics and evaluation of loss-to follow-up

	Whole sample	No follow-up	Follow-up	*p *value
N	1377	260	1117	
Gender (female)	58.7	53.1	60.0	0.042
Age, mean (SD)	47.0 (13.5)	45.0 (13.6)	47.5 (13.5)	0.008
Education (high vocational/ university)	60.4	57.3	61.1	0.261
Radiating pain into the leg	39.9	41.9	39.4	0.453
Radiating pins and needles	27.0	26.5	27.1	0.848
Time since complaints 1st started	11.4 (12.0)	11.2 (11.7)	11.4 (12.1)	0.792
Duration of current episode (years)	2.1 (4.8)	2.4 (4.8)	2.1 (4.8)	0.423
SF-6D baseline score	0.74 (0.11)	0.75 (0.11)	0.74 (0.11)	0.691
ODI baseline score	23.4 (15.7)	24.6 (16.6)	23.2 (15.5)	0.265
Concomitant complaints present	53.8	54.2	53.7	0.881
Pain avoidant	70.9	72.8	70.5	0.490
Previous specialist visit	62.1	57.3	63.2	0.078
Previous visit neurologist	22.5	25.0	21.9	0.287
Previous visit orthopaedic surgeon	21.2	23.1	20.8	0.413
Previous visit rehabilitation	4.9	6.2	4.7	0.317
Previous visit pain clinic	7.5	6.5	7.7	0.522
Medication (categorical)*				0.477
* None (ref.)*	32.8	28.8	33.8	
* Rarely*	24.5	25.0	24.4	0.327
* Regularly not daily*	28.2	30.8	27.7	0.139
* Daily*	14.5	15.4	14.2	0.280
Previous physiotherapy*				0.987
* Treated no effect (ref.)*	64.6	65.0	64.5	
* Treated effect*	6.6	6.5	6.6	0.939
* Not treated*	28.8	28.5	28.9	0.875
Previous manual therapy*				0.899
* Treated no effect (ref.)*	28.8	29.2	28.7	
* Treated effect*	6.4	5.8	6.5	0.649
* Not treated*	64.8	65.0	64.7	0.934
Previous chiropractic*				0.034
* Treated no effect (ref.)*	13.6	18.5	12.4	
* Treated effect*	2.6	3.5	2.8	0.136
* Not treated*	83.8	79.6	84.4	0.013
Previous medication*				0.735
* Treated no effect (ref.)*	19.5	18.5	19.7	
* Treated effect*	2.8	3.5	2.7	0.440
* Tot treated*	77.7	78.1	77.6	0.691
Previous pain clinic*				0.046
* Treated no effect (ref.)*	6.0	3.8	6.5	
* Treated effect*	0.9	1.9	0.6	0.015
* Not treated*	93.1	94.2	92.8	0.114
Previous surgery*				0.333
* Treated no effect (ref.)*	1.7	1.9	1.7	
* Treated effect*	2.0	0.8	2.2	0.181
*Tot treated*	96.3	97.3	96.1	0.829
Previous other treatment*				0.789
* Treated no effect (ref.)*	16.6	15.8	16.8	
* Treated effect*	4.4	3.8	4.6	0.783
* Not treated*	78.9	80.4	78.6	0.643
Work status*				0.126
* No work (ref.)*	22.3	22.7	22.3	
* Not physical work*	51.6	46.5	52.6	0.423
* Physical work*	26.1	30.8	25.1	0.331
Type of treatment*				0.064
* Manual Medicine (MM, ref.)*	20.0	21.4	19.7	
* Orthomanual Medicine (OMM)*	76.9	74.0	77.6	0.461
* Both MM and OMM*	1.8	1.3	1.8	0.529
* Other treatment*	1.3	3.1	0.9	0.024
Number of treatment sessions	3.4 (1.6)	2.7 (1.6)	3.5 (1.6)	0.000
McKenzie	14.2	12.6	14.6	0.434
Treated.differently	4.4	5.4	4.2	0.428

For dichotomous and categorical variables percentages are presented, for continuous variables means and standard deviation (SD). Loss to follow-up is evaluated for each baseline variable with *p *values for the probability to be included in the analyses (patients completing at least one follow-up measurement)

*For categorical variables reference categories are indicated in the table

### Missing data loss to follow-up

Additional file 1: Table S1 shows the handling of predictor variables, including the percentages of missing values. Only one variable, Baseline ODI, showed a high percentage of missing values (25.6%), which could be explained by the tailored distribution of the ODI to patients who had self-classified as LBP patient. Because not all LBP patients classified themselves as such, not all LBP patients received the ODI. Because the percentage of missing Baseline ODI values was > 20, it was decided to impute the baseline ODI.

Evaluation of loss to follow-up is presented in Table 1. It shows that baseline scores on the ODI, SF6D, and NRS did not differ significantly between patients who only answered the baseline questionnaire and the patients included in the analyses. Female patients (p = 0.042), older patients (p = 0.008), and patients treated effectively by a chiropractor (p = 0.034) were significantly more inclined to remain in our study. Patients treated effectively at a pain clinic were significantly less inclined to remain in our study (p = 0.046).

### Defining subgroups with distinct pain trajectories

**Figure 1:**
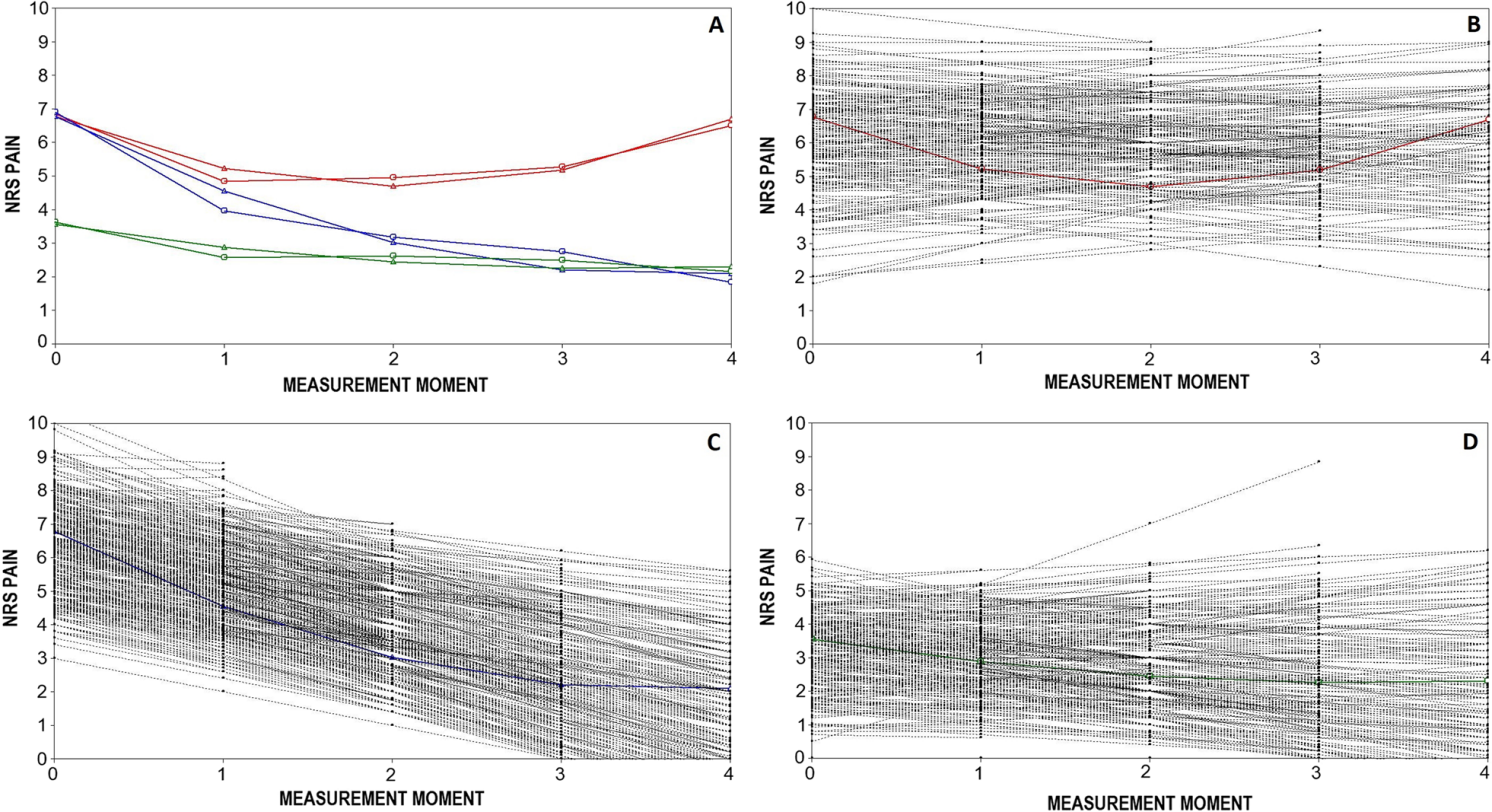
**A**–**D** Mean estimated trajectories (**A**) and separate trajectories per class, with individual trajectories of all patients (**B**–**D**). The X-axis represents the measurement moments (1 = baseline, 2 = 6 weeks, 3 = 12 weeks, 4 = 18 weeks, and 5 = 26 weeks follow-up). The Y-axis represents the NRS mean group scores (red line: not improved patients (26.5%), blue line: improved patients (46.0%), green line: patients with low baseline pain levels (27.5%)). In (**A**), squared lines represent sample trajectories, triangular lines represent estimated trajectories. **B**–**D** show the estimated trajectory with the individual trajectories of all individual patients for not improved patients (**B**), improved patients (**C**), and patients with low baseline NRS (**D**) separately. Grey lines represent the individual trajectories of all patients

Model fit characteristics are presented in Additional file 1: Table S2. Although a four-class quadratic model without fixed variance showed slightly better fit (BIC 17,836 versus 17,842), it was decided to choose the three-class quadratic model without fixed variance as the preferred model, because of its better interpretability and practicality. Posterior probabilities of the three class model were slightly better than the posterior probabilities of the four class model (mean posterior probabilities ranged from 0.769–0.854 for the three class model versus 0.717–0.848 for the four class model). Furthermore, the four-class model included a small group of patients (7.0%) who showed a strong improvement in the first three months, and a return to previous pain levels in the subsequent months. Compared to the three-class model, this for a large part only changed the proportion of patients in the group that started with high pain levels and showed no improvement, suggesting that this group consisted of patients who remained unchanged during the study combined with patients who improved strongly, only to deteriorate again. Figure 1A-D shows the mean estimated trajectories (Fig. 1A), and the three separate trajectories together with the individual trajectories of all patients for class1 (Fig. 1B), class2 (Fig. 1C), and class 3 (Fig. 1D). The course of the average NRS scores of the three groups is presented in Table 2, together with the mean changes in the ODI scores. In the three-class model, a group of 226 patients started with high NRS scores at baseline and showed hardly any change during the follow-up period (mean NRS-pain changed from 6.9 to 6.7). In this group the mean ODI score changed from 24.8 to 19.4. A group of 578 patients started with high baseline scores and showed considerable improvement (mean NRS changed from 7.0 to 1.8). In this group, the mean ODI score changed from 26.4 to 6.1. A group of 313 patients started with lower baseline scores and showed moderate, but clinically relevant improvement (mean NRS changed from 3.5 to 2.2). In this group the mean ODI score changed from 15.5 to 8.0.

**Table 2:** Mean NRS and ODI scores of the three classes

Mean NDI and ODI	Class 1 (N = 226, 20%)		Class 2 (N = 578, 52%)		Class 3 (N = 313, 28%)	
	NRS (SD)	ODI (SD)	NRS (SD)	ODI (SD)	NDI (SD)	ODI (SD)
Baseline	6.9 (1.2)	24.8 (15.8)	7.0 (1.1)	26.4 (15.4)	3.5 (1.1)	15.5 (12.7)
6 weeks	5.2 (2.2)	17.6 (14.5)	4.0 (2.4)	15.0 (14.4)	2.5 (1.9)	8.6 (9.7)
12 weeks	5.7 (2.4)	19.7 (15.6)	2.9 (2.3)	11.0 (11.4)	2.6 (2.2)	8.7 (10.8)
18 weeks	5.7 (2.1)	18.9 (16.3)	2.6 (2.2)	8.1 (10.2)	2.5 (2.2)	8.6 (10.4)
26 weeks	6.7 (1.2)	19.4 (14.7)	1.8 (1.4)	6.1 (7.3)	2.2 (1.7)	8.0 (9.9)
Baseline-26 wk. change	0.2 (3%)	5.4 (22%)	5.2 (74%)	20.3 (77%)	1.3 (37%)	7.5 (48%)

Mean NRS and ODI scores (SD) of all three LCGA classes at baseline and at all follow-up moments, and the score change between baseline and 26 weeks follow-up

### Predictors

In Table 3 the baseline characteristics for the three groups of patients with distinct pain trajectories identified with LCGA are presented. Because the group of patients with low baseline pain scores could be identified by the baseline NRS scores, our main interest was to identify predictors that distinguish patients with high NRS scores who showed a favourable course from patients with high NRS scores who did not show a favourable outcome. Further analyses therefore focused on the two subgroups that started with high pain scores, i.e. the group of patients that was considered to be improved and the group of patients that was considered to be not improved. Baseline variables were evaluated as possible predictors of a favourable course. For all baseline variables, univariate odds ratio’s for improvement are presented. The relationship between the continuous predictors and group membership was shown to be linear for all continuous variables except for the duration of the current episode, which was further analysed as a spline variable. In this spline variable, two stages could be identified. If the duration of the current episode was less than four years, the odds of a favourable outcome decreased with a longer duration. If the duration of the current episode was longer than four years, the odds of a favourable outcome hardly changed. Male gender, previous specialist visit, previous surgical treatment, and having work were associated with a favourable course. Previous consultation with a neurologist or an orthopaedic surgeon, no effect of previous treatments and concomitant complaints were associated with a non-favourable outcome. Other baseline variables did not show a significant association with the outcome.

**Table 3:** Characteristics of three groups of patients with different pain trajectories and univariate probability for improvement

Predictor variables	LCGA classes of 1117 patients classified			Univariate analyses			
	Class 1	Class 2	Class 3	Class 2 versus Class 1			
	Not improved	Improved	Low NRS	*p* value	OR	95% CI of OR	
N	226	578	313			Lower	Upper
Gender (female)	30.1	42.2	43.1	0.002	0.589	0.424	0.818
Age, mean (SD)	47.6 (14.4)	47.4 (13.8)	47.5 (12.2)	0.845	0.999	0.988	1.010
Education (high vocational/ university)	60.1	57.8	67.9	0.448	0.883	0.641	1.217
Radiating pain into the leg	43.8	42.9	29.7	0.817	0.964	0.707	1.315
Radiating pins and needles	31.0	28.0	22.7	0.407	0.932	0.788	1.102
Time since complaints 1st started	11.6 (12.3)	11.4 (12.2)	11.4 (11.6)	0.855	0.999	0.986	1.011
Duration of current episode (years)	3.2 (6.2)	1.9 (4.8)	1.7 (3.6)	0.003	0.958	0.932	0.986
SF-6D baseline score	0.73 (0.11)	0.73 (0.10)	0.78 (0.10)	0.523	0.613	0.137	2.746
ODI baseline score	24.8 (15.8)	26.4 (15.4)	15.5 (12.7)	0.525	1.004	0.992	1.015
Concomitant complaints present	58.8	51.4	54.3	0.048	0.731	0.535	0.998
Pain avoidant	73.5	73.6	62.1	0.938	0.986	0.697	1.397
Previous specialist visit	52.2	64.2	69.3	0.002	1.640	1.201	2.240
Previous visit neurologist	28.3	20.9	19.2	0.026	0.819	0.687	0.976
Previous visit orthopedic surgeon	28.8	19.7	16.9	0.006	0.847	0.753	0.953
Previous visit rehabilitation	7.1	5.0	2.2	0.255	0.912	0.779	1.068
Previous visit pain clinic	8.8	8.5	5.4	0.866	0.991	0.888	1.105
Medication (categorical)							
None (ref.)	26.1	28.9	48.2	0.231			
Rarely	23.9	24.6	24.3	0.738	0.929	0.603	1.430
Regularly not daily	35.4	28.4	20.8	0.113	0.724	0.486	1.080
Daily	14.6	18.2	6.7	0.640	1.124	0.688	1.837
Previous physiotherapy							
Treated no effect (ref.)	72.6	63.5	60.4	0.037			
Treated effect	3.1	5.9	10.5	0.069	2.170	0.943	4.998
Not treated	24.3	30.6	29.1	0.044	1.438	1.009	2.049
Previous manual therapy							
Treated no effect (ref.)	35.4	27.3	26.5	0.066			
Treated effect	4.9	6.7	7.3	0.112	1.795	0.873	3.692
Not treated	59.7	65.9	66.1	0.036	1.429	1.024	1.994
Previous chiropractic							
Treated no effect (ref.)	20.4	11.1	9.3	0.003			
Treated effect	2.7	2.9	2.6	0.165	2.036	0.746	5.563
Not treated	77.0	86.0	88.2	0.001	2.053	1.354	3.113
Previous medication							
Treated no effect (ref.)	27.0	21.3	11.5	0.224			
Treated effect	2.2	2.4	3.5	0.546	1.389	0.478	4.033
Tot treated	70.8	76.3	85.0	0.086	1.367	0.957	1.952
Previous pain clinic							
Treated no effect (ref.)	10.2	6.2	4.5	0.152			
Treated effect	0.9	0.7	0.3	0.787	1.278	0.216	7.548
Not treated	88.9	93.1	95.2	0.055	1.710	0.989	2.957
Previous surgery							
Treated no effect (ref.)	4.4	0.9	1.3	0.010			
Treated effect	2.2	2.1	2.6	0.040	4.800	1.074	21.447
Tot treated	93.4	97.1	96.2	0.003	5.318	1.797	15.739
Previous other treatment							
Treated no effect (ref.)	19.5	15.6	17.3	0.369			
Treated effect	4.0	3.5	7.0	0.851	1.086	0.457	2.581
Not treated	76.5	81.0	75.7	0.171	1.323	0.886	1.974
Work status							
No work (ref.)	31.4	21.1	17.9	0.005			
Not physical work	48.2	51.4	58.1	0.013	1.586	1.100	2.286
Physical work	20.4	27.5	24.0	0.002	2.012	1.296	3.122
Type of treatment							
Manual Medicine (MM, ref.)	14.6	21.5	18.6	0.109			
Orthomanual Medicine (OMM)	70.4	76.5	77.7	0.166	0.739	0.482	1.133
Both MM and OMM	2.2	0.9	3.1	0.058	0.284	0.078	1.042
Other treatment	0.4	1.1	0.7				
Number of treatment sessions	3.7 (1.5)	3.5 (1.6)	3.5 (1.6)	0.121	0.925	0.839	1.021
McKenzie	11.1	13.3	18.2	0.801	1.065	0.645	1.733
Treated.differently	6.1	4.6	2.1	0.008	1.000	1.000	1.000

Baseline characteristics for the three groups of patients with different trajectories separately, and results of the univariate analyses of improved versus not improved patients (*p* values, odds ratio’s and 95% confidence intervals). For dichotomous variables percentages are presented, for continuous variables mean and (SD)

### Multivariable model

A multivariable model was constructed, and the model without treatment variables is presented in Table 4. In this model male gender, previous specialist visit, previous pain clinic treatment, having work, a shorter duration of the current episode, and a longer time since the complaints first started were predictive of a favourable course. No effect of previous chiropractic treatment was predictive of a non-favourable course, both compared with patients reporting a positive effect of previous chiropractic treatment, or patients not previously treated by a chiropractor. The fitted model showed an AUC of 0.671, with a non-significant Hosmer and Lemeshow test (0.732), supporting model fit, and the amount of information in the model (R^2^) was 0.10. Bootstrap validation resulted in a corrected R^2^ of 0.06. Adding treatment variables in the backwards selection analysis resulted in a model which included the number of treatments (OR 0.91), but no other treatment variables such as the type of SMT administered or adjuvant treatment with McKenzie. The fit of this model was practically the same as the fit of the model without treatment variables (AUC 0.677, Hosmer and Lemeshow 0.734, R^2^ 0.10, and bootstrap R^2^ 0.06).

**Table 4:** Multivariable model

Predictor variables in multivariate model	Coefficient	OR	95% CI of OR	
			Lower	Upper
Gender (female)	− 0.6271	0.5341	0.3629	0.7862
Time since complaints 1st started	0.0145	1.0146	0.9982	1.0312
Previous specialist visit	0.4623	1.5876	1.0645	2.3678
Previous visit pain clinic	0.1372	1.1471	0.9982	1.3162
Previous chiropractic treatment (treated without effect is reference)				
Treated effective	0.2836	1.3279	0.4244	4.1546
Not treated	0.5804	1.7868	1.0872	2.9364
Work status (no work is reference)				
Non-physical work	0.3539	1.4246	0.9218	2.2014
Physical work	0.5169	1.6768	0.9900	2.8399
Duration of current episode in years (non-linear spline variable)				
Duration of current episode*	− 0.5197	0.5947	0.4298	0.8228
Spline variable duration of current episode*	1.9619	7.1128	1.9705	25.6750
Intercept	0.5462	1.7266	0.8101	3.6801

Multivariable model without treatment variables using baseline variables to predict a favourable outcome in patients presenting with high NRS for pain scores, including the Odds ratio’s (OR) and the 95% Confidence Intervals of the OR

*The Odds Ratio of the spline variables cannot be interpreted, but a longer duration of the current episode decreased the probability of improvement up to a period of four years and remained stable thereafter

## Discussion

Studying the clinical course of low back pain in patients consulting MSK physicians in The Netherlands with Latent Class Growth Analyses, three distinct pain trajectories were identified. More than half of all the patients (52%) presented with high baseline pain scores and showed considerable improvement during six months follow-up. A second group of patients with high baseline pain scores (20% of all patients) showed no improvement. A third group, with moderate baseline pain scores (28%) showed slight, but clinically relevant improvement. The multivariable model presented showed a moderate discrimination, with an AUC of 6.77, and poor calibration, with an R^2^ of 0.10. Therefore one can question its usefulness in clinical practice. Apparently, with the baseline data collected in our study, it is hard to predict which patient might improve after consulting an MSK physician.

Previous studies almost invariably reported similar clusters of pain trajectories, generally including clusters with persistent high pain, clusters with more or less persistent moderate or low pain, clusters showing improvement, and clusters with a fluctuating pattern. The proportion of patients in each cluster, however, differed, possibly because of variations in the study designs. Patients were recruited in General Practice [6–8, 27], at chiropractic clinics [10, 28], combined in General Practice and Chiropractic clinics [29], combined in General Practice and physiotherapy practices [30], or in a population based survey [31]. Also, studies varied in recruiting patients: i.e. only patients with chronic [30], only acute [27], or a mix of both chronic and acute LBP [6–8, 10, 28, 29, 31]. Moreover, follow-up periods varied from 12 weeks [27] to one year [10, 29–31], and follow-up measurements varied form weekly text messages [10, 28, 29] to monthly questionnaires [6–8]. The population based study was the only study in which a cluster showing improvement was not reported [31]. And the only study recruiting acute LBP patients showed high percentages of recovery [27]. The clusters presented in our study are well comparable to those reported in other studies, with the exception of a cluster representing a fluctuating pattern. In our four class model a small cluster was added that would in other studies have qualified as fluctuating. We considered this cluster merely a subgroup of the consistent high pain cluster, eventually showing no improvement after six months follow-up, and therefor chose to use the three class model.

Most trajectory studies reported variables that were associated with group membership. Although varying variables were reported, only higher pain intensity [6–8, 27], longer duration [6, 7, 27, 29, 32], and more physical disability [8] were more or less consistently associated with a more severe trajectory. The same variables were reported in other studies to be associated with a worse prognosis in LBP patients, together with unemployment [33, 34]. Similarly, in our study the duration of the current episode and unemployment were both associated with a lower probability of improvement, but baseline disability was not associated with the outcome. In our univariate analysis, ineffective previous treatments were consistently predictive of an unfavourable course. Of these previous treatments reported in our study, only chiropractic treatment ended up in our prediction model.

Our study used pain trajectories to identify different courses of LBP after MSK treatment, rather than use a singular outcome measurement. Groups of patients with different trajectories were compared to assess whether baseline variables could predict a possible favourable course. We would consider that the different trajectories thus identified represent a more relevant estimation of whether patients have improved or not. A challenging question remains to what extent the clinical course represented the natural recovery or the consequence of the treatment administered. A multivariable model including treatment variables only included the number of treatments sessions, but not the type of SMT, or the adjuvant use of McKenzie treatment. No conclusions can be drawn from these findings because patients were not randomized, and physicians were at liberty to choose the type of treatment. The higher number of treatment sessions in the group of unimproved patients, for example, may well have been influenced by elongating the treatment in patients who reported no improvement to their treating physician.

### Strengths and limitations

A strength of our study was the web-based data-collection, which enabled us to follow a large number of patients at a relatively low cost. In this way data could be collected from patients who consulted an MSK physician, with questionnaires that were tailored to their main complaint. A weakness was the difficulty to identify low back pain patients before consulting the physician. Our solution using web-based self-classification, aided with manikins, appeared to lead to a high proportion of miss-classification. Because we tailored the distribution of PROMs to the main complaint as reported by the patient, this miss-classification led to a high percentage of missing baseline ODI values. We therefore chose to use the physician’s diagnosis to identify patients with LBP, and we imputed the baseline ODI. Another weakness of our study set-up was the high proportion of patients that discontinued their participation. The response rate gradually diminished during the follow-up period. Out of the 1117 patients included in the baseline population 93% responded after 6 weeks, 74% after 12 weeks, 58% after 18 weeks, and 43% at six months. We found that some baseline variables were related to loss to follow-up which made the MAR assumption more plausible, supporting multiple imputation of missing values. However, loss to follow-up may have had an impact on the long-term course of the pain trajectories identified. Another limitation is the inclusion of all patients completing at least one follow-up measurement in our analyses. The fact that patients frequently missed intermediate questionnaires, however, supports the assumption that these measurements are missing at random.

## Conclusion

In patients with low back pain, three different clinical courses were identified in the six months after consulting an MSK physician in the Netherlands. A large group of patients presented with high baseline pain scores, and showed improvement. In patients with a high pain score at baseline, a multivariable prediction model showed a number of predictors of a favourable course. In this model, male gender, longer time since the complaints first started, shorter duration of the current episode of pain, previous specialist visit, previous pain clinic visit, effective treatment by a chiropractor, or no previous chiropractic treatment, and having work were predictors of a favourable course. The prediction model, however, showed a low AUC and a low amount of information. It is a continuing challenge to identify predictors of a favourable outcome in LBP patients.

## Supplementary Information

**Additional file 1: Table S1.** Handling of predictor variables, **Table S2.** Fit characteristics of pain trajectories.

## Data Availability

The datasets and more detailed information about statistical analyses generated during the current study are not publically available due to possible privacy conflicts, but are available upon reasonable request.
