# Supplementary material for: Pain trajectories and possible predictors of a favourable course of low back pain in patients consulting musculoskeletal physicians in The Netherlands
Source: Chiropr Man Therap. 2021 Sep 22;29:38. doi: 10.1186/s12998-021-00392-3 (PMC8456566; doi:10.1186/s12998-021-00392-3)
Supplement: Supplementary file 1 — Additional file 1: Table S1. Handling of predictor variables, Table S2. Fit characteristics of pain trajectories. [file 12998_2021_392_MOESM1_ESM.docx]

Table S1. Handling of predictor variables.

| Predictor | Type | Missing % | Handling | Values/ remarks |
| --- | --- | --- | --- | --- |
|  |  |  |  |  |
| Gender | dichotomous | 0 | unchanged | Male/female |
| Age | continuous | 0 | unchanged | none |
| Education | categorical | 1.8 | dichotomized | higher/lower education |
| Radiating pain into the leg | dichotomous | 0 | unchanged | Yes/ no |
| Radiating pins and needles | dichotomous | 0 | unchanged | Yes/ no |
| Time since start 1st complaints | continuous | 0.6 | unchanged | none |
| Duration current pisode | continuous | 3.9 | unchanged | none |
| Baseline SF-6D | continuous | 0.4 | unchanged | none |
| Baseline ODI | continuous | 25.6 | imputed | none |
| Previous specialist visit | dichotomous | 0 | unchanged | Yes/no |
| Previous visit neurologist | dichotomous | 0 | unchanged | Yes/no |
| Previous visit orthopedic | dichotomous | 0 | unchanged | Yes/no |
| Previous visit rehabilitaion | dichotomous | 0 | unchanged | Yes/no |
| Previous visit pain clinic | dichotomous | 0 | unchanged | Yes/no |
| Medication | categorical | 0 | unchanged | Four categories; none, rarely, regularly not daily, daily |
| Concomitant complaints | dichotomous | 0 | unchanged | Yes/no |
| Previous physiotherapy | dichotomous | 0 | categorized | Combined into one categorical value, treated without  effect (reference), treated with effect, or not treated |
| Effect physiotherapy | ordinal |  |  |  |
| Previous manual therapy | dichotomous | 0 | categorized | Combined into one categorical value, treated without  effect (reference), treated with effect, or not treated |
| Effect manual therapy | ordinal |  |  |  |
| Previous chiropractic treatment | dichotomous | 0 | categorized | Combined into one categorical value, treated without  effect (reference), treated with effect, or not treated |
| Effect chiropractic treatment | ordinal |  |  |  |
| Previous medication | dichotomous | 0 | categorized | Combined into one categorical value, treated without  effect (reference), treated with effect, or not treated |
| Effect medication treatment | ordinal |  |  |  |
| Previous pain clinic treatment | dichotomous | 0 | categorized | Combined into one categorical value, treated without  effect (reference), treated with effect, or not treated |
| Effect pain clinic | ordinal |  |  |  |
| Previous surgical treatment | dichotomous | 0 | categorized | Combined into one categorical value, treated without  effect (reference), treated with effect, or not treated |
| Effect surgical treatment | ordinal |  |  |  |
| Previous treatment other | dichotomous | 0 | categorized | Combined into one categorical value, treated without  effect (reference), treated with effect, or not treated |
| Effect other treatment | ordinal |  |  |  |
| Work status | various dich.  variables | 0 | categorized | Combined into one categorical variable, no work  (reference),not physical work and physical work |
| Type of work |  |  |  |  |
| Pain avoidant | continuous | 0 | dichotomized | Pain avoidant yes/ no (no; FABQ <14) |
| Type of treatment | various dich.  variables | 7.9 | categorized | Combined into one categorical value, MG treatment  (reference), OMG treatment, both, or none |
| Number of treatment sessions | continuous | 7.9 | unchanged | none |
| McKenzie therapy | dichotomous | 7.9 | unchanged | Yes/no |
| Treated differently | various dich.  variables | 7.9 | dichotomized | Combined into one dichotomous variable, treated  differently yes/ no |

Table S2. LCGA models; Several models were evaluated with 1-5 classes, quatratic or linear, and with subgroup variance fixed to 0 or free. Model fit was evaluated with the Vuong-Lo-Mendell-Rubin likelihood ratio test (LMR-LRT) and the Bayesian Information Criterion (BIC). The Akaike information criterion (AIC) is presented as well. The choice of the best model was based upon model fit, clinical interpretability and practicality.

| Model | 1 | 2 | 3 | 4 | 5 | 6 | 7 | 8 | 9 | 10 | 11 | 12 | 13 | 14 | 15 | 16 |
| --- | --- | --- | --- | --- | --- | --- | --- | --- | --- | --- | --- | --- | --- | --- | --- | --- |
| Classes | 2 | 3 | 4 | 5 | 2 | 3 | 4 | 5 | 2 | 3 | 4 | 5 | 2 | 3 | 4 | 5 |
| Quadratic | Yes | Yes | Yes | Yes | No | No | No | No | No | No | No | No | Yes | Yes | Yes | Yes |
| Variance | 0 | 0 | 0 | 0 | 0 | 0 | 0 | 0 | Free | Free | Free | Free | Free | Free | Free | Free |
| Likelihood | -8948 | -8888 | -8850 | -8830 | -9156 | -9125 | -9097 | -9084 | -9106 | -9091 | -9077 | -9067 | -8885 | -8844 | -8826 | -8806 |
| AIC | 17923 | 17810 | 17743 | 17711 | 18333 | 18276 | 18226 | 18207 | 18239 | 18214 | 18193 | 18178 | 17807 | 17732 | 17705 | 17672 |
| BIC | 17988 | 17896 | 17849 | 17836 | 18383 | 18341 | 18306 | 18302 | 18304 | 18295 | 18288 | 18228 | 17898 | 17842 | 17836 | 17822 |
| p-value* | 0.000 | 0.002 | 0.000 | 0.000 | 0.000 | 0.147 | 0.000 | 0.056 | 0.000 | 0.065 | 0.173 | 0.018 | 0.000 | 0.000 | 0.029 | 0.074 |
| Interpretability | + | +++ | + | - | + | +++ | + | - | + | +++ | + | - | + | +++ | + | - |

* p-value based upon the LMR-L
